# Pilot Study of Quality-of-Life Differences Between Surgical and Non-Surgical Organ Preservation Strategies in Head and Neck Cancer Patients

**DOI:** 10.7759/cureus.100728

**Published:** 2026-01-04

**Authors:** Zoi Zachou, Dimitrios Dimitroulis, Ourania Natsiopoulou, Zoi Gartagani, Panagiotis Chadoulos, Ioannis Seggas, Efthymios Kyrodimos

**Affiliations:** 1 School of Medicine, National and Kapodistrian University of Athens, Athens, GRC; 2 Second Department of Propaedeutic Surgery, Laiko Hospital, National and Kapodistrian University of Athens, Athens, GRC; 3 Hellenic Minimally Invasive and Robotic Surgery Study Group, National and Kapodistrian University of Athens, Athens, GRC; 4 First Department of Otorhinolaryngology, Hippocration General Hospital of Athens, Athens, GRC

**Keywords:** functional outcomes, head neck cancer, quality of life, survivors, treatment strategies

## Abstract

Introduction

Head and neck cancer (HNC) treatments can significantly affect long-term quality of life, with different treatment modalities impacting other aspects of patient well-being. Our study aimed to evaluate quality of life (QoL), functional outcomes, and symptom impact in head and neck cancer (HNC) patients and to compare outcomes between patients who underwent definitive surgical treatment and those treated with non-surgical organ preservation therapy.

Materials and methods

A pilot cohort study was performed between September 2024 and September 2025 in Hippocrateion General Hospital, Athens, Greece, including 25 HNC patients diagnosed and treated with curative intent. Participants completed the European Organisation for Research and Treatment of Cancer Quality of Life Questionnaire Core 30 (EORTC QLQ-C30) and Quality of Life Questionnaire-Head and Neck 35 (QLQ-H&N35) (Greek versions) at least three months after their treatment. Statistical analysis was performed using SPSS, and p-values <0.05 were considered significant.

Results

From the 25 participants, 13 had surgical treatment, and 12 underwent non-surgical organ preservation therapy (radiotherapy ± chemotherapy). The mean global QoL score was 59.3, higher in the surgical group than in the chemoradiotherapy group (65.36 vs. 52.73, p=0.15). Functional outcomes were good overall, with a mean functional score of 77.84 and slightly better results in the surgical group (81.05 vs. 74.35). Fatigue was the most common symptom (mean score 41.72), particularly in the chemoradiotherapy group (48.1 vs. 35.83, p 0.18). In the QLQ-H&N35 questionnaire, painkiller use, sensory loss (taste and smell), and sexual problems were the main problems in surgical patients. In contrast, xerostomia and weight loss were the main concerns among the non-surgical organ preservation group.

Conclusions

Patients from both treatment groups had satisfactory QoL; however, symptom profiles differed between the groups. Surgical patients used painkillers more commonly and had more sensory disturbances, impacting their quality of life. In contrast, those treated with non-surgical organ preservation therapy had more common fatigue, problems with xerostomia, and weight loss. These modality-specific challenges should be considered in personalised survivorship to optimise post-treatment recovery and good quality of life in HNC patients.

## Introduction

Head and neck cancer (HNC) is the seventh most frequently occurring cancer worldwide [1]. With the advances in oncologic treatment, current 5-year survival rates now exceed 60%-80% [2,3] for localised and locally advanced disease [3]. Consequently, the population of Head and Neck Cancer (HNC) survivors, people with or beyond cancer who are still living, continues to grow [4]. In HNC survivors, both surgery and chemoradiotherapy (CRT) significantly impact oral function and quality of life (QoL) [5]. The acute side effects of treatment may persist beyond treatment, while additional chronic effects may develop at least 90 days after treatment discontinuation [6]. As a result, patients remain at risk of many significant short and long-term side effects, including mucositis, xerostomia, taste changes, altered smell perception, lymphedema, osteonecrosis, hypothyroidism, dysphagia, hearing loss, shoulder dysfunction, neuropathic pain, chronic fatigue, and malnutrition [6-9].

One of the most prevalent and debilitating side effects of HNC treatment is dysphagia [10]. This debilitating sequela can affect survivors’ functionality, quality of life (QoL), mental and emotional well-being, as well as financial stability. Perhaps in no other group of cancer patients does QoL present as important a role as in HNC patients [11]. Quality of life is a multidimensional subjective parameter, requiring a scale covering a wide range of items in a variety of fields (physical, cognitive, etc)[12].

Specific scales and instruments are designed to evaluate particular conditions, such as the European Organisation for Research and Treatment of Cancer Quality of Life Questionnaire Core 30 (EORTC QLQ-C30) for cancer, and the Quality of Life Questionnaire-Head and Neck 35 (QLQ-H&N35) or the Functional Assessment of Cancer Therapy-Head and Neck (FACT-H&N) for head and neck malignancies. Other tools focus on specific clinical domains or symptoms, including fatigue, depression, xerostomia, and dysphagia, or on particular treatment modalities such as surgery and radiotherapy. Because these instruments are tailored to capture condition-related and treatment-related changes with greater sensitivity, they are employed in the majority of studies involving head and neck cancer populations [13,14].

The literature regarding QoL is not rich, and the existing literature is more focused on post-radiotherapy impact on QoL [11,13-15] and less on surgical patients [6], while in this group, research is more focused on specific aspects of surgical techniques, mainly the use of flaps [16,17]. The comparison between organ preservation non-surgical treatment (RT or CRT) and surgery with curative intent is scarce. Our study primarily aims to assess QoL across all domains in head and neck cancer survivors and to compare QoL between patients who received organ preservation therapy and those who had definitive surgical treatment.

## Materials and methods

Study design

A pilot cohort study was conducted from September 2024 until September 2025, including 25 patients with head and neck cancer. The patients were diagnosed at Hippocrateion General Hospital in Athens, Greece, a tertiary centre, and, if surgical treatment was required, it was performed there by experienced Head and Neck surgeons. A post-treatment assessment was made with widely used, validated HNC questionnaires; the EORTC QLQ-C30 (version 3) [18,19] and the EORTC QLQ-H&N35 [20] module in the Greek language. The assessment was made at least three months after treatment to evaluate its impact on quality of life, thereby minimizing the influence of acute post-treatment toxicities.

Bioethics

Ethical approval to use human participants was obtained from the Hippokrateion General Hospital Research Ethics Committee (Protocol Number 10667). A consent form was obtained from all the participants of the study. Permission from the EORTC to download and use the questionnaires in the Greek language and the scoring manuals was obtained (request ID 118444).

Participants

The inclusion criteria for study participation were a) HNC patients with squamous cell carcinoma (SCC) histologically confirmed in larynx, oropharynx, oral cavity, hypopharynx and unknown primary, b) age >18 years old, c) patients treated with curative intent, d) patients who received organ preservation therapy or surgical treatment, and e) assessment >3 months post-therapy. Exclusion criteria included other histology types, skin cancer, rare sites such as salivary glands, and palliative treatment, as stated by the participants and their records.

Statistical methods

SPSS (IBM SPSS Statistics, version 22**; **IBM Corp., Armonk, NY, USA) was used for statistical analysis. Normality was assessed with the Shapiro-Wilk test, and the Mann-Whitney U test was used to compare the groups. Statistically significant results were considered those with p<0.05.

## Results

A total of 25 participants were included in the study, of whom 13 underwent surgical treatment with no need for post-operative adjuvant treatment, and 12 received non-surgical organ preservation therapy with radiotherapy ± concurrent chemotherapy with curative intent. The mean age was 69.77 years (Standard Deviation (SD) 11.8, range: 44-83 years). The majority (80%) of patients were male in both groups, while 20% were female. The most common primary tumour site was the larynx in both groups (68%). The demographic characteristics are presented in detail in Table 1.

**Table 1:** Participants' demographics.

Patients’ characteristics	N	Percent (%)
Gender		
Male	20	80
Female	5	20
Primary Site		
Larynx	17	68
Oropharynx	3	12
Oral Cavity	2	8
Other	3	12
Treatment		
Non-surgical organ preservation treatment (RT±CT)	12	48
Surgery	13	52

A detailed scoring in all scales for the EORTC QLQ-C30 is described in Table 2. Normality in all domains was tested with the Shapiro-Wilk test, with domains with p<0.05 not normally distributed. Normality is assumed in QoL, physical functioning, emotional functioning, fatigue and speech. Not normal distribution was assumed in functional scales, role functioning, cognitive functioning, social functioning, symptom scales, nausea, pain, dyspnea, insomnia, appetite loss, constipation, diarrhoea, financial difficulties, pain, swallowing, teeth, opening mouth, dry mouth, sticky saliva, senses, cough, felt ill, social eating, sexuality, painkillers, nutritional supplements, feeding tube, weight loss and weight gain. The Mann-Whitney U test was used for group comparisons due to non-normal distributions in the majority of domains and the small sample size. The overall quality of life (QoL) among head and neck cancer survivors was above average, with a mean global QoL score of 59.3. Patients who underwent surgical treatment reported a higher QoL than those who received radiotherapy and possible concurrent chemotherapy, but the difference was not statistically significant (65.36 vs. 52.73, respectively, p=0.15).

**Table 2:** Score of the EORTC QLQ-C30 version 3.0 in each section. To compare the means between the two groups, the Mann-Whitney U test was used.

EORTC QLQ-C30 (version 3.0)	Score M±SD	Non-surgical organ preservation Treatment Score M±SD	Surgical treatment Score M±SD	Statistical Significance p<0.05	Mann-Whitney U
Global health status/QoL	59.3 ± 26.60	52.73 ± 26.18	65.36 ± 26.54	0.15	51.00
Functional scales	77.84 ±18.12	74.35 ± 19.24	81.05 ± 17.15	0.40	62.50
Physical functioning	75.96 ± 20.91	70.51 ± 26.13	81.00 ± 13.84	0.50	65.50
Role functioning	69.98 ±41.66	62.49 ± 47.2	76.90 ± 36.35	0.65	69.00
Emotional functioning	71.96 ± 22.56	70.77 ± 21.49	73.05 ± 24.33	0.53	66.00
Cognitive functioning	87.98 ±16.34	87.47 ± 12.58	88.44 ± 19.71	0.50	65.50
Social functioning	83.31±27.23	80.53 ± 29.16	85.88 ± 26.23	0.50	65.50
Symptom scales / items	24.72 ±16.95	26.83 ± 18.52	22.77 ± 15.86	0.81	73.50
Fatigue	41.72 ±27.81	48.1 ± 30.44	35.83 ± 24.89	0.18	53.50
Nausea and vomiting	2.07 ±7.46	4.15 ± 10.34	0.00 ± 0.00	0.51	60.00
Pain	19.41± 27.6	21.15 ± 25.87	17.93 ± 30.01	0.64	63.00
Dyspnoea	33.2 ± 33.3	38.75 ± 31.19	28.07 ± 35.58	0.16	52.00
Insomnia	33.30 ± 31.06	33.30 ± 34.79	33.30 ± 28.39	0.93	70.00
Appetite loss	23.59 ± 31.79	27.75 ± 27.80	19.43 ± 36.10	0.34	55.50
Constipation	33.30 ± 32.58	27.75 ± 27.80	38.86 ±37.14	0.55	61.00
Diarrhoea	11.99 ± 25.22	19.43 ± 33.19	5.12 ± 12.50	0.40	62.00
Financial difficulties	26.64 ± 31.90	22.2 ± 25.92	30.75 ± 37.16	0.72	71.00

Regarding functional outcomes, the mean score on the functional scale was 77.84, indicating a high level of functionality across the cohort. Both treatment groups demonstrated good functional status, with higher scores in the surgical group (81.05 vs. 74.35). The highest scores were observed in the cognitive functioning domain in both groups. Symptom burden was generally low, as reflected by a mean symptomatology score of 24.72 in QLQ-C30. Both treatment groups had low symptom levels, with the surgical group reporting fewer complaints (22.77 vs. 26.83). Among the assessed general symptoms with the QLQ-C30, nausea and vomiting were the least concerning, while fatigue was the most frequently reported problem in both groups, with a mean score of 41.72, higher in the non-surgical organ preservation group (48.1 vs. 35.83, p = 0.18).

A detailed scoring in all symptom scales for the EORTC QLQ-H&N35 is shown in Table 3. In the QLQ-H&N35, the most frequently reported problem was the need for painkillers, despite the pain scores themselves being relatively low. The need for painkillers was more pronounced in the surgical group (mean score 53.84) than in the radiotherapy ± chemotherapy group (mean score 41.66) (p = 0.61).

**Table 3:** Score of the EORTC QLQ-H&N35 in each section. To compare the means between the two groups the Mann-Whitney U test was used.

EORTC QLQ-H&N35	Score M±SD	Non-surgical organ preservation Treatment Score M±SD	Surgical treatment Score M±SD	Statistical Significance p<0.05	Mann-Whitney U
Symptom scales					
Pain	18.47 ± 25.23	28.21 ± 32.11	9.48 ± 12.07	0.13	50.5
Swallowing	21.59 ± 30.59	29.77 ± 37.65	14.04 ± 21.07	0.37	61.50
Teeth	23.88 ± 33.98	24.83 ± 32.06	23.00 ± 36.95	0.72	71.00
Opening mouth	11.92 ± 26.87	16.5 ± 26.32	7.69 ±27.73	0.34	60.00
Dry mouth	27.66 ± 38.84	48.36 ± 45.61	10.15 ± 20.80	0.04	36.5
Sticky saliva	24.87 ± 36.99	38.75 ± 42.21	11.00 ±25.69	0.08	42.00
Senses	33.08 ± 30.00	23.33 ± 27.94	42.07 ±30.02	0.07	45.50
Coughing	37.04 ± 32.21	33.16 ± 37.55	40.61 ±27.45	0.43	63.50
Felt ill	19.92 ± 35.92	27.66 ± 39.71	12.76 ±31.93	0.32	59.00
Speech	35.68 ± 27.46	34.91 ± 32.95	36.38 ±22.62	0.57	67.00
Social eating	17.03 ± 27.83	27.27 ± 35.96	7.58 ±12.75	0.20	54.50
Social contact	17.46 ± 22.08	14.81 ± 19.07	19.9 ±25.07	0.65	69.00
Sexuality	40.43 ± 43.79	40.08 ± 41.75	40.81 ±47.97	0.88	63.50
Pain killers	48.00 ± 50.99	41.66 ± 51.49	53.84 ±51.88	0.61	68.50
Nutritional supplements	16.00 ± 37.41	25.00 ± 45.22	7.69 ±27.73	0.47	64.50
Feeding tube	20.83 ± 41.48	27.27 ± 46.70	15.38 ± 37.55	0.64	63.00
Weight loss	36.00 ± 48.98	50.00 ± 52.22	23.07 ± 43.85	0.27	57.00
Weight gain	20.00 ± 40.82	16.66 ± 38.92	23.07 ±43.85	0.81	73.00

Among patients who underwent surgery, the main concerns, apart from the need for pain medication, were loss of senses (taste and smell), with a mean score of 42.07, followed by sexuality-related problems. In contrast, patients in the chemoradiotherapy group reported weight loss (mean score 50) as their most severe symptom, followed by dry mouth (mean score 48.36). Dry mouth and sticky saliva were more frequently reported in the non-surgical organ preservation group, and dry mouth showed a statistically significant difference between the two groups (p=0.04). The least troublesome issue for the surgical group was social eating, with a mean score of 7.58, and in the non-surgical organ-preservation group, mouth opening (score 16.5).

## Discussion

This study evaluated the quality of life (QoL), functional outcomes, and symptom impact on head and neck cancer (HNC) survivors who underwent either surgical or non-surgical organ preservation therapy. Overall, patients demonstrated good QoL, with a mean score of 59.3, consistent with literature indicating that most HNC survivors adapt well post-treatment. Orozco et al (2025) report that they rate their health-related QoL as “good” after one year of follow-up [21], and Gomes et al (2020) also reported a QLQ-C30 QoL score of 61.62 [22].

In our study, patients who underwent surgical treatment reported higher QoL scores than those who received radiotherapy +/- chemotherapy; however, no statistical significance was found (p=0.15). This finding is in accordance with earlier research suggesting that while radiotherapy offers organ preservation, it is often associated with chronic toxicities, such as xerostomia, taste alterations, and swallowing difficulties, that significantly impair QoL [23,24]. Radiation-induced salivary gland dysfunction, a dose-dependent effect, leads to persistent xerostomia in up to 80% of survivors, consequently reducing oral comfort and social participation. These late effects may explain the lower QoL scores observed in the organ preservation group in our study. Surgical patients reported better global QoL but greater impairment in taste and smell, consistent with previous findings that surgical interventions involving upper aerodigestive structures often affect sensory recovery and neural regeneration [25].

Taste and smell loss were among the most significant reported concerns, while other domains, such as speech and swallowing, which are believed to be the main issues, scored lower, likely reflecting advances in reconstructive and rehabilitative strategies. The use of painkillers was highly scored in both groups, being the central issue in surgical patients; however, the pain was not a chief complaint, leading to the conclusion that it is well managed in these patients. Pain in head and neck cancer can be chronic, severe, and persistent despite completion of oncologic treatment, as described in the literature. Treatments for head and neck cancer have the potential to increase pain and discomfort. In radical surgical treatment, significant structural changes may lead to pain. Radiation therapy may cause late fibrosis of skin and soft tissues that may lead to temporomandibular joint dysfunction and myofascial pain syndromes [26].

Both groups showed good functional outcomes, with the surgical group scoring slightly higher on the functional scale. The literature in this field is scarce, with some studies reporting poor functional outcomes after radiotherapy [27]. Our participants’ high functional scores suggest that early rehabilitative interventions and multidisciplinary follow-up may have lessened these sequelae. Cognitive functioning, which showed the highest subscale scores in both groups, possibly reflects preserved psychosocial adaptation, consistent with prior evidence that acceptance of altered QoL improves with increasing age and time since treatment [21].

The overall symptom burden was low, aligning with reports that HNC survivors generally exhibit stable or improving symptom profiles over time. Fatigue was the most frequently reported general symptom in both groups, findings also confirmed in prior studies where tiredness, anxiety, and drowsiness remained the most common persistent complaints [28].

In the head and neck-specific module (QLQ-H&N35), the leading symptoms differed by treatment group. Surgical patients most often reported painkiller use, sensory loss and sexual problems, whereas chemoradiotherapy patients experienced severe xerostomia, a well-recognised symptom as a chronic sequelae of radiation therapy [24] and weight loss. In literature, surgery and especially total laryngectomy impacted both patients and partners with negative effects being reported in more than 30% of cases. The physical transformation has esthetic and emotional impact, inhibiting sexuality [29]. Dry mouth was found to be statistically significant between groups, which aligns with the literature on radiation-induced salivary dysfunction [23,24]. Persistent salivary dysfunction not only causes dryness but also contributes to dysgeusia, dysphagia, and social eating difficulties [30], which in turn could lead to weight loss.

Our findings highlight the importance of individualised survivorship care, emphasising symptom management, sensory rehabilitation, and fatigue reduction. Depending on the treatment plan, subsequent support could be tailored to each group’s specific needs. While non-surgical organ preservation strategies aim to maintain function, their chronic side effects, especially xerostomia and fatigue, need proactive management through salivary-sparing radiation techniques, hydration strategies, and fatigue-specific interventions. Surgical survivors may benefit more from sensory retraining and psychosocial support to address body image and sexual well-being.

In our study, the small sample size limits generalizability. Future research with longitudinal follow-up, larger cohorts, and integration of patient-reported outcome measures could provide deeper insight into the evolving QoL of HNC survivors.

## Conclusions

In summary, both surgical and organ preservation approaches can achieve good QoL and functionality in head and neck cancer survivors, although symptom patterns partially differ by treatment modality. Persistent issues such as fatigue, pain management, xerostomia, and sensory loss warrant continued multidisciplinary attention to enhance survivorship outcomes and overall well-being. Our findings emphasise the importance of individualised treatment planning and long-term supportive care to optimise both clinical outcomes and quality of life for head and neck cancer patients.

## References

[1] Sun H, Yu M, An Z, Liang F, Sun B, Liu Y, Zhang S (2025). Global burden of head and neck cancer: epidemiological transitions, inequities, and projections to 2050. Front Oncol.

[2] Liao LJ, Hsu WL, Lo WC, Cheng PW, Shueng PW, Hsieh CH (2019). Health-related quality of life and utility in head and neck cancer survivors. BMC Cancer.

[3] Gormley M, Creaney G, Schache A, Ingarfield K, Conway DI (2022). Reviewing the epidemiology of head and neck cancer: definitions, trends and risk factors. Br Dent J.

[4] Tota JE, Best AF, Zumsteg ZS, Gillison ML, Rosenberg PS, Chaturvedi AK (2019). Evolution of the oropharynx cancer epidemic in the united states: moderation of increasing incidence in younger individuals and shift in the burden to older individuals. J Clin Oncol.

[5] Armache M, Larson A, Stemme R (2025). Novel survivorship paradigms in head/neck cancer. Semin Radiat Oncol.

[6] Yokoi A, Maruyama T, Yamanaka R, Takeuchi N, Morita M, Ekuni D (2024). Relationship among cancer treatment, quality of life, and oral function in head and neck cancer survivors: A cross-sectional study. Support Care Cancer.

[7] Tashimo Y, Ihara Y, Yuasa K (2019). Acute stage longitudinal change of quality of life from pre- to 3 months after surgical treatment in head and neck cancer patients. Asian Pac J Cancer Prev.

[8] Brook I (2020). Late side effects of radiation treatment for head and neck cancer. Radiat Oncol J.

[9] Lou DI, Dietrich MS, Deng J, Murphy BA (2021). Mechanisms of pain and their manifestations in head and neck cancer: Importance of classifying pain subtypes. Head Neck.

[10] Huynh TM, Falk RS, Hellebust TP (2024). Chronic fatigue in long-term survivors of head and neck cancer treated with radiotherapy. Radiother Oncol.

[11] Ihara Y, Crary MA, Madhavan A, Gregorio DC, Im I, Ross SE, Carnaby GD (2018). Dysphagia and oral morbidities in chemoradiation-treated head and neck cancer patients. Dysphagia.

[12] Wan Leung S, Lee TF, Chien CY, Chao PJ, Tsai WL, Fang FM (2011). Health-related quality of life in 640 head and neck cancer survivors after radiotherapy using EORTC QLQ-C30 and QLQ-H&amp;N35 questionnaires. BMC Cancer.

[13] Heutte N, Plisson L, Lange M, Prevost V, Babin E (2014). Quality of life tools in head and neck oncology. Eur Ann Otorhinolaryngol Head Neck Dis.

[14] Chuang EY, Hou PY, Shueng PW (2024). Improved quality of life in head and neck cancer patients treated with modern arc radiotherapy techniques - A prospective longitudinal analysis. Front Oncol.

[15] Crowder SL, Douglas KG, Yanina Pepino M, Sarma KP, Arthur AE (2018). Nutrition impact symptoms and associated outcomes in post-chemoradiotherapy head and neck cancer survivors: a systematic review. J Cancer Surviv.

[16] Agea Martínez M, Antúnez-Conde R, Navarro Cuéllar C (2022). Assessment of quality of life in head-and-neck oncologic patients with intraoral soft-tissue defects reconstructed with buccinator myomucosal flap. J Clin Med.

[17] Lahtinen S, Molnár K, Hietanen S, Koivunen P, Ohtonen P, Alakärppä A, Liisanantti J (2022). Quality of life in head and neck cancer patients at 5 years after free flap reconstruction: a significant decline during the follow-up. Eur Arch Otorhinolaryngol.

[18] Aaronson NK, Ahmedzai S, Bergman B (1993). The european organization for research and treatment of cancer QLQ-C30: a quality-of-life instrument for use in international clinical trials in oncology. J Natl Cancer Inst.

[19] Fayers PM, Aaronson NK, Bjordal K (2001). The EORTC QLQ-C30 Scoring Manual (3rd Edition). Manual (3rd Edition).Published by: European Organisation for Research and Treatment of Cancer, Brussels.

[20] Bjordal K, Hammerlid E, Ahlner-Elmqvist M (1999). Quality of life in head and neck cancer patients: validation of the European Organization for Research and Treatment of Cancer Quality of Life Questionnaire-H&amp;N35. J Clin Oncol.

[21] Orozco-Núñez S, Oishi N, Izquierdo J, Martínez-Expósito F, García-LLiberós A, Zapater E (2025). Postoperative evolution of health-related quality of life in patients with head and neck cancer. J Surg Oncol.

[22] Gomes EP, Aranha AM, Borges AH, Volpato LE (2020). Head and neck cancer patients’ quality of life: analysis of three instruments. J Dent (Shiraz).

[23] Nathan CO, Asarkar AA, Entezami P (2023). Current management of xerostomia in head and neck cancer patients. Am J Otolaryngol.

[24] Kramer B, Wenzel A, Boerger M (2019). Long-term quality of life and nutritional status of patients with head and neck cancer. Nutr Cancer.

[25] McLaughlin L (2013). Taste dysfunction in head and neck cancer survivors. Oncol Nurs Forum.

[26] Chua KSG, Reddy SK, Lee MC (1999). Pain and loss of function in head and neck cancer survivors. J Pain Symp Manag.

[27] Obeso-Benítez P, Muñoz-Vigueras N, Castillo-Pérez I, Rodríguez-Torres J, Granados-Santiago M, Cabrera-Martos I, Valenza MC (2022). Global functional impairment in head and neck cancer survivors after completing radiotherapy treatment. Disabil Rehabil.

[28] Gostian M, Allner M, Rak A (2024). Symptom burden and quality of life in long-term survivors with head and neck cancer. Ear Nose Throat J.

[29] Babin E, Heutte N, Humbert M, Laccourreye O (2023). Sex-related quality of life after total laryngectomy for cancer. Eur Ann Otorhinolaryngol Head Neck Dis.

[30] Funk GF, Karnell LH, Christensen AJ (2012). Long-term health-related quality of life in survivors of head and neck cancer. Arch Otolaryngol Head Neck Surg.

